# Triterpenoid Contents and Anti-Inﬂammatory Properties of the Methanol Extracts of *Ligustrum* Species Leaves

**DOI:** 10.3390/molecules16010001

**Published:** 2010-12-23

**Authors:** Chi-Rei Wu, You-Cheng Hseu, Jin-Cherng Lien, Li-Wei Lin, Yung-Ta Lin, Hui Ching

**Affiliations:** 1 Graduate Institute of Chinese Pharmaceutical Sciences, College of Pharmacy, China Medical University, 91 Hsieh Shih Road, Taichung 40402, Taiwan; 2 Department of Cosmeceutics, College of Pharmacy, China Medical University, 91 Hsieh Shih Road, Taichung 40402, Taiwan; E-Mail: ychseu@mail.cmu.edu.tw (Y.-C.H.); 3 Graduate Institute of Pharmaceutical Chemistry, College of Pharmacy, China Medical University, 91 Hsieh Shih Road, Taichung, 40402 Taiwan; E-Mail: jclien@mail.cmu.edu.tw (J.-C.L.); 4 The School of Chinese Medicines for Post-Baccalaureate, I-Shou University, No.8, Yida Rd., Yanchao Township, Kaohsiung County 82445, Taiwan; E-Mail: lwlin@isu.edu.tw (L.-W.L.); 5 Department of Pharmacy, Taichung Tzu Chi General Hospital, No.66, Fongsing Rd., Tanzih Township, Taichung County 427, Taiwan; E-Mail: pharmacy54321@hotmail.com (Y.-T.L.); 6 Taichung Hospital, Department of Health, The Executive Yuan, Taichung 40402, Taiwan; E-Mail: hui235911@mail.tbcnet.net (H.C.)

**Keywords:** *Ligustrum* plants, *Ligustrum pricei*, analgesic activity, anti-inﬂammatory activity, triterpenoids

## Abstract

*Ligustrum* (privet) plants are used by Chinese physicians to prevent and cure hepatitis and chronic bronchitis. Three common *Ligustrum* plant spp., namely *Ligustrum lucidum* Ait. (LL), *L. pricei* Hayata (LP) and *L. sinensis* Lour. (LS) were collected to assess their analgesic/anti-inflammatory properties on chemical-induced nociception and carrageenan-induced inflammation in rodents. The methanol extracts from *Ligustrum* plants leaves effectively inhibited nociceptive responses induced by 1% acetic acid and 1% formalin. LP and LL reduced the edema induced by 1% carrageenan. LP exhibited the best potency of the *Ligustrum* plants. Furthermore, LP reduced the abdominal Evan’s blue extravasations caused by lipopolysaccharide, lipoteichoic acid, autocrines and sodium nitroprusside. The triterpenoid content of the three *Ligustrum* spp. was measured by high performance liquid chromatography using a photodiode array detector. LP contained the highest content of amyrin, betulinic acid and lupeol. LL had the highest content of oleanolic acid and ursolic acid. The various degrees of analgesic/anti-inflammatory effects among three *Ligustrum* plants may be related to their different triterpenoid contents. LP is a potential analgesic and anti-inflammatory *Ligustrum* plant. The effects of LP are partially related to the inhibition of cyclooxygenase-2 activity and a decrease in microvascular permeability via the actions of autocrines and kinins.

## 1. Introduction

Plants of the genus *Ligustrum* (privet, Oleaceae) are traditionally used in Chinese medicine to prevent and cure hepatitis and chronic bronchitis. *Ligustrum lucidum* Ait. (abbreviated as LL), a major type of *Ligustrum* plant, possesses anti-inflammatory, antibacterial, hepatoprotective and antidiabetic activities [1,2,3,4]. *L. pricei* Hayata (LP) and *L. sinensis* Lour. (LS) are other *Ligustrum* species commonly cultivated in Southeast Asia and also used as other sources of *Ligustrum* medicines. However, no scientific report regarding the *in vivo* and *in vitro* anti-inflammatory and analgesic activities of LP and LS has been published. Consequently we have now evaluated the analgesic and anti-inflammatory properties of methanol extracts of these *Ligustrum* species in an acetic acid-induced writhing test [5], the formalin-induced licking test [6] and the carrageenan-induced paw edema test [7]. Moreover we also clarified the anti-inflammatory mechanism of LP using a dermal microvascular permeability test that measured Evan’s blue dye extravasations induced by the bacterial cell wall components lipopolysaccharide (LPS), lipoteichoic acid (LTA) and some inflammatory mediators, such as serotonin, histamine, bradykinin and sodium nitroprusside (SNP). 

**Figure 1:**
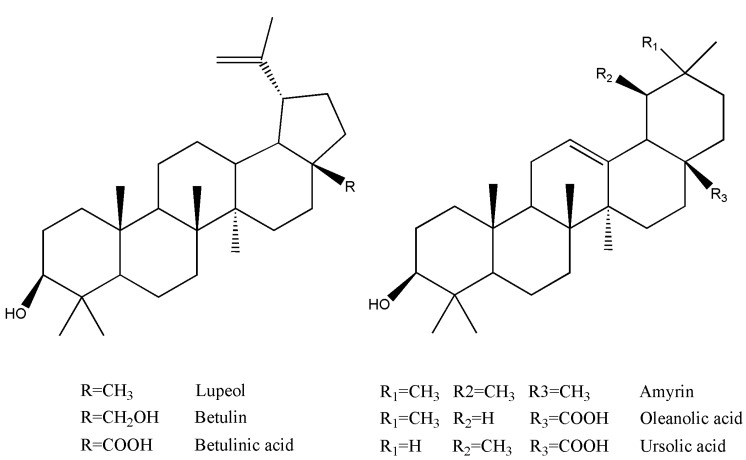
Structures of the six assayed triterpenoids.

Oleanolic acid and ursolic acid are the major active components responsible for LL’s hepatoprotective, antidiabetic and antibacterial activities [1,3,4]. Therefore, we collected samples of these *Ligustrum* plants and assayed their contents of six common triterpenoids, including betulin, betulinic acid, oleanolic acid, ursolic acid, amyrin and lupeol (Figure 1), using high performance chromatography equipped with a photodiode array detector (HPLC-PDA).

## 2. Results and Discussion

### 2.1. Analgesic activity of methanol extract from Ligustrum plants leaves in mice

Chemical-induced visceral pain and paw nociception are very useful models for the study of nociception and the assessment of analgesic drugs [8]. In the present study, two widely accepted and different mechanistic experimental nociceptive models, the acetic acid-induced abdominal writhing response and the formalin-induced paw licking response, were used to evaluate the analgesic properties of methanol extracts of *Ligustrum* plant leaves. In the acetic acid-induced nociceptive test, the writhing number of mice pretreated with vehicle, methanol extracts from the *Ligustrum* plants leaves (0.1, 0.25, 1 g/kg) and the positive control ASA (0.3 g/kg) are shown in Table 1. The methanol extracts from the *Ligustrum* plants leaves at 0.25 and 1 g/kg decreased the acetic acid-induced writhing number in a dose-dependent manner (*p* < 0.01, *p* < 0.001). The inhibition percentage caused by LP, LS and LL at 1 g/kg in the acetic acid-induced writhing response varied from 38.8 to 57.7% with the highest inhibiting activity being observed for LP. The positive control ASA at 0.3 g/kg also decreased acetic acid-induced writhing number with an inhibition of 42.8 % (*p* < 0.01) (Table 1). 

**Table 1:** Effects of methanol extract from *Ligustrum* plants leaves (0.1, 0.25, 1 g/kg) on acetic acid-induced writhing response in mice.

Groups	Dose (g/kg)	Average writhing	Percentages of protection
VEH		42.3 ± 1.9	-
*L. pricei*	0.1	42.2 ± 2.4	0
	0.25	28.0 ± 0.9**	33.8
	1	17.9± 1.9***	57.7
*L. sinense*	0.1	43.0 ± 1.1	-1.7
	0.25	30.8 ± 2.0**	27.2
	1	19.3 ± 1.9***	54.4
*L. lucidum*	0.1	39.4 ± 4.2	6.9
	0.25	31.3 ± 3.6*	26
	1	25.9 ± 2.6**	38.8
ASA	0.3	24.2 ± 2.1**	42.8

Data are expressed as mean ± SEM for eight mice each group. ** *p* < 0.01, *** *p* < 0.001 compared with VEH group.

**Figure 2:**
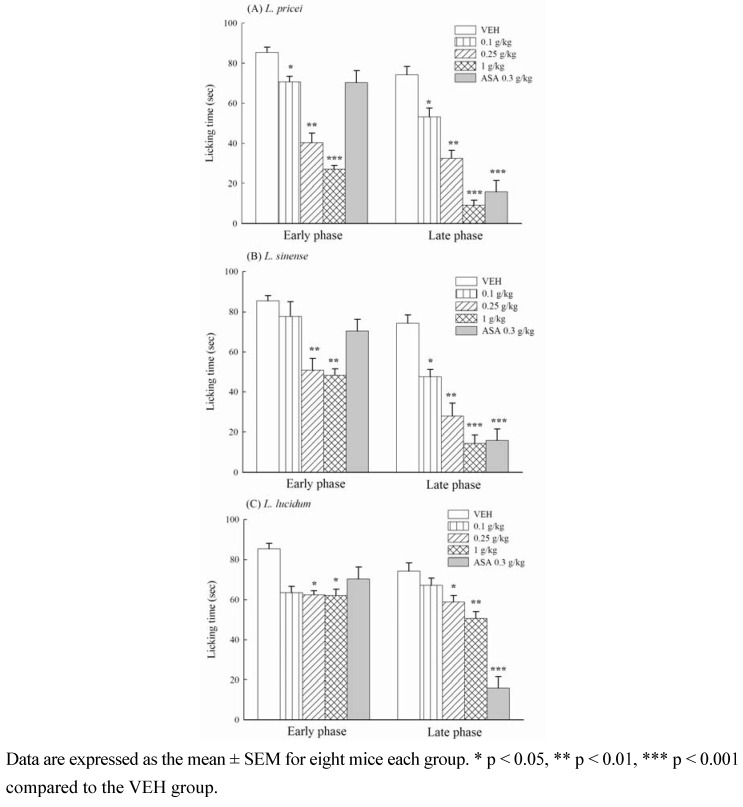
Effects of methanol extracts from *Ligustrum* plants leaves (0.1, 0.25, 1 g/kg) and acetylsalicylic acid (ASA, 0.3 g/kg) on the formalin-induced licking response in mice.

In the formalin-induced nociceptive test, the licking time of mice pretreated with vehicle (VEH group), methanol extracts from the *Ligustrum* plant leaves (0.1, 0.25, 1 g/kg) and the positive control ASA (0.3 g/kg) are shown in Figure 2. The methanol plant leave extracts shortened the licking time induced by formalin during the early and late phase in a dose-dependent manner (*p* < 0.05, *p* < 0.01, *p* < 0.001). The inhibition percentage caused by LP, LS and LL at 1 g/kg on the formalin-induced licking response varied from 27.2 to 68.3% in the early phase and from 31.8 to 87.6% in the late phase. LP exhibited the highest inhibiting activity in the biphasic phase of formalin-induced licking response*.* The positive control ASA at 0.3 g/kg inhibited the late, but not the early phase of the formalin-induced licking response in mice with a maximal inhibition of 78.6% (*p* < 0.001) (Figure 2).

The present data provided that the methanol extracts from *Ligustrum* plants leaves possess analgesic activities in a dose-dependent manner. The methanol extracts at 0.25 g/kg inhibited the acetic acid-induced abdominal writhing response and the two phases of formalin-induced licking response in mice. The results are similar to all of the previous research reports for other *Ligustrum* plants [9]. These analgesic dose of the methanol extracts from *Ligustrum* plants leaves were from 0.25 g/kg and lower than those of other *Ligustrum* plant that the ED_50_ of *L. robustum* on the analgesic effect is 1.7 g/kg and the analgesic dose of its purified fraction must be 0.5 g/kg [10,11]. However, the positive control, ASA, also decreased the acetic acid-induced writhing response but only decreased the late phase of formalin-induced licking response. The result of ASA on the acetic acid-induced writhing response and the formalin-induced licking response are consistent with our previous report and a series of reports by Shibata *et al.* [6,12]. Because the acetic acid-induced abdominal writhing response is primarily based on the peripheral system [13], and there are differential central and peripheral properties in the formalin-induced biphasic licking responses [6], we suggest that the analgesic property of *Ligustrum* plants leaves is different than the analgesic produced by ASA. *Ligustrum* plants leaves from 0.25 to 1 g/kg possess central analgesic and peripheral analgesic/anti-inflammatory properties in mice. LP has the best analgesic activity of three commonly used *Ligustrum* plants and other *Ligustrum* plant [10,11].

**Figure 3:**
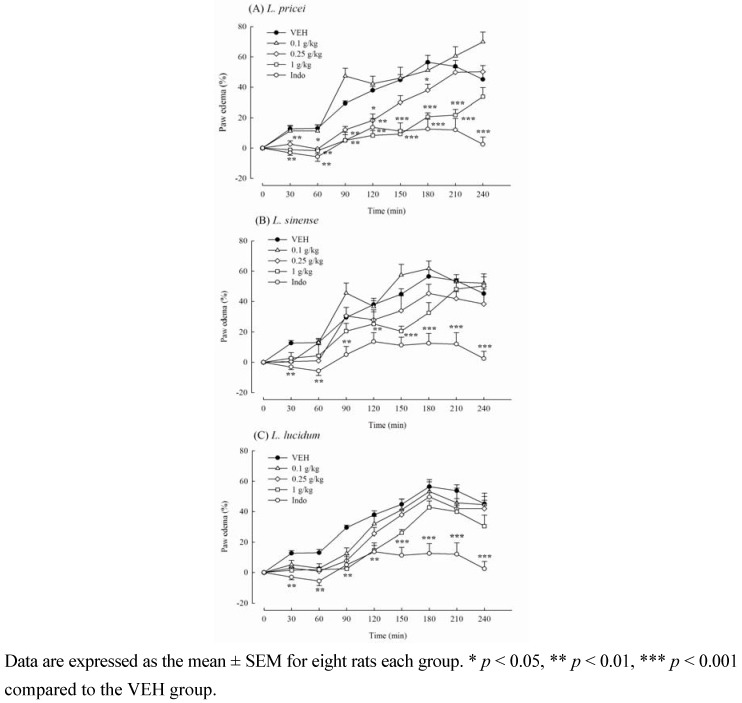
Effects of methanol extracts from *Ligustrum* plants leaves (0.1, 0.25, 1 g/kg) and indomethacin (Indo, 10 mg/kg) on carrageenan-induced paw edema in mice.

### 2.2. Anti-inflammatory activity of methanol extract from the Ligustrum plants leaves in rats

Due to the inhibitory activities of the methanol extract from *Ligustrum* plants leaves on the inflammatory algesia (late phase) of formalin-induced licking responses, we further assessed the anti-inflammatory activity of the methanol extract from *Ligustrum* plants leaves against carrageenan-induced edema formation in rats. The paw edema percentage caused by 1% carrageenan at 1, 2, 3, and 4 h was 13.06 ± 2.22, 37.91 ± 2.60, 56.42 ± 4.66, and 45.15 ± 4.83, respectively. Pretreatment with the methanol extract from the *Ligustrum* plants leaves revealed that only LP from 0.25 to 1 g/kg and LL at 1 g/kg decreased paw edema level caused by 1% carrageenan (Figure 3). 

LP (1 g/kg) significantly decreased the edema percentage to -1.71 ± 2.06, 8.33 ± 3.30, and 20.69 ± 2.57 at 1, 2 and 3 h after carrageenan treatment, respectively (*p* < 0.01, *p* < 0.001). LL (1 g/kg) significantly decreased the edema percentage to 1.90 ± 1.36 and 14.56±3.24 at 1 and 2 h after carrageenan treatment, respectively (*p* < 0.01). The positive control Indo (10 mg/kg) also decreased carrageenan-induced paw edema percentage to -5.74 ± 3.03, 13.66 ± 5.93, 12.53 ± 6.52, and 2.46 ± 4.75 at 1, 2, 3, and 4 h after carrageenan treatment, respectively (*p* < 0.01, *p* < 0.001) (Figure 3). Therefore, we suggested that LP is a potential analgesic and anti-inflammatory *Ligustrum* plant among the three commonly used *Ligustrum* plants.

### 2.3. Effects of the methanol extract from LP leaves on the microvascular permeability in rats

**Figure 4:**
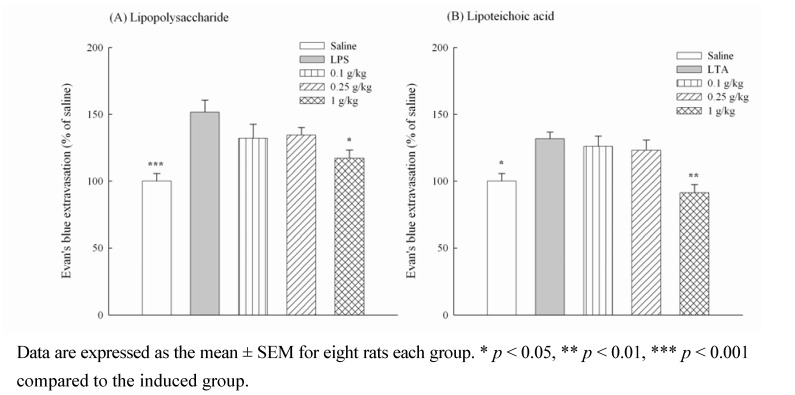
Effects of methanol extracts from *Ligustrum pricei* (0.1, 0.25, 1 g/kg) on microvascular permeability increased by lipopolysaccharide (LPS, A) and lipoteichoic acid (LTA, B) in rats.

Bacterial infections are involved in several inflammatory diseases. LPS is the major etiologic component of pathogenic Gram-negative bacteria. LPS stimulates host cells and leads to severe inflammatory responses induced by Gram-negative bacterial infection. Unlike Gram-negative bacteria, Gram-positive bacteria lack LPS and instead contain LTA on their cell wall. Increasing reports have indicated that LTA acts, similar to LPS, as a central inducer of the inflammatory responses and plays a role in the pathogenesis of severe inflammatory responses induced by Gram-positive bacterial infection. Because LL inhibits periodontal pathogen and the inflammatory response caused by LPS *in vitro* [2,4], further investigation of LP from 0.25 to 1 g/kg on the microvascular permeability of the inflammatory cascade produced by LPS and LTA with Evan’s blue dye extravasations was performed in rats. The abdominal Evan’s blue dye extravasations in the marked circle that received intradermal saline represented 100%. The percentages of abdominal Evan’s blue dye extravasations increased to 151.65 ± 8.78 and 131.74 ± 8.78 when rats were intradermally administered with the Gram negative bacteria cell wall component, LPS, or Gram positive bacteria cell wall component, LTA, respectively (Figure 4). LP at 1 g/kg decreased the percentage of abdominal Evan’s blue dye extravasations increased by LPS (*p* < 0.05) and LTA (*p* < 0.01) (Figure 4). The inflammatory cascade and edema formation caused by LPS or LTA are mediated by many inflammatory mediators, including autocrines, kinins and prostaglandins, which lead to a dilation of arterioles and venules and to an increase in microvascular permeability [14,15]. Moreover, carrageenan-induced edema usually separates into three phases. The first phase, 1.5 h after carrageenan treatment, is related to autocrines and platelet activating factors. The second phase, from 1.5 h to 2.5 h after carrageenan treatment, is related to kinins. The third phase, 2.5 h after carrageenan treatment, is related to prostaglandins and leukotriens [16,17].

**Figure 5:**
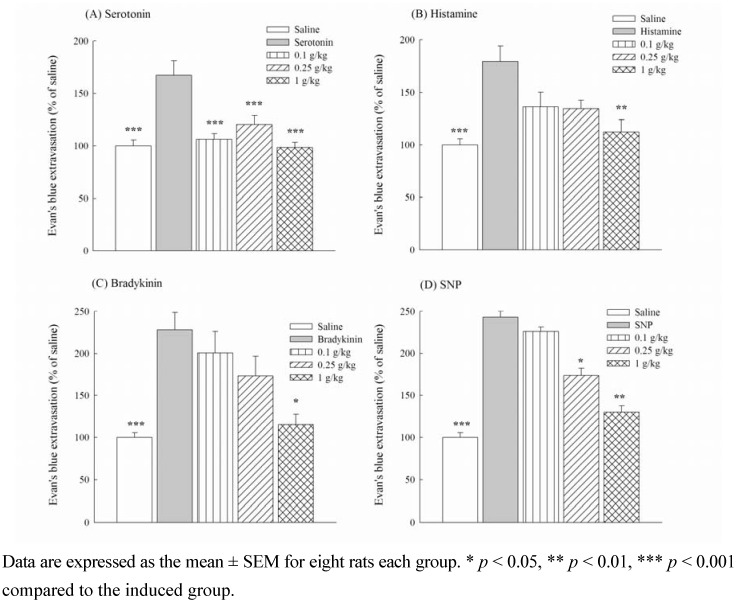
Effects of methanol extracts from *Ligustrum pricei* (0.1, 0.25, 1 g/kg) on microvascular permeability increased by serotonin (A), histamine (B), bradykinin (C) and SNP (D) in rats.

However, Shibata *et al*. suggested that substance P and bradykinin are involved in the early phase of the formalin-induced licking responses, and autocrines, bradykinin and prostaglandin participate in the late phase [6]. Therefore, to further clarify the anti-inflammatory mechanism of LP from 0.25 to 1 g/kg, microvascular permeability induced by autocrines, bradykinin and SNP was measured with Evan’s blue dye extravasations in rats. The percentages of abdominal Evan’s blue dye extravasations increased to 167.28 ± 13.95, 179.67 ± 14.71, 228.04 ± 20.81 and 243.06 ± 17.81 when rats were intradermally administered with inflammatory mediators such as serotonin, histamine, bradykinin, and SNP, respectively (Figure 5). LP from 0.25 to 1 g/kg decreased the percentage of abdominal Evan’s blue dye extravasations caused by serotonin (*p* < 0.001), but at only 1 g/kg significantly decreased the percentage of abdominal Evan’s blue dye extravasations caused by histamine and SNP (*p* < 0.01) and bradykinin (*p* < 0.05) (Figure 5). Therefore, the anti-inflammatory effects of LP from 0.25 to 1 g/kg on the formalin-induced licking response and carrageenan-induced paw edema might be related to the modulation of inflammatory mediators, including prostaglandins, nitric oxide, autocrines and kinins.

### 2.4. The cyclooxygenase-2 inhibiting activities of methanol extract from Ligustrum plants leaves in vitro

Because LL inhibits the inflammatory response caused by LPS via NF-kappaB and cyclooxygenase-2 pathway *in vitro* [2,4], we further evaluated the cyclooxygenase-2 inhibiting activities of methanol extracts from *Ligustrum* plants leaves to demonstrate their analgesic/anti-inflammatory property *in vitro*. The IC_50_ values of the methanol extracts from *Ligustrum* plants leaves (LL, LP and LS) against cyclooxygenase-2 activity were 485.49 ± 30.49, 94.83 ± 1.66 and 231.66 ± 7.48 μg/mL, respectively. The result was consistent with the analgesic potency of LP, which possesses the better inhibitory effects against cyclooxygenase-2 activity than LS and LL*.* The analgesic and anti-inflammatory mechanism of LP, also similar to the report of LL [2], might be partially related to the inhibition of the biosynthesis of inflammatory mediators, such as prostaglandins, via its cyclooxygenase-2 inhibitory activity.

### 2.5. Triterpenoid contents of methanol extract from Ligustrum plants leaves

**Figure 6:**
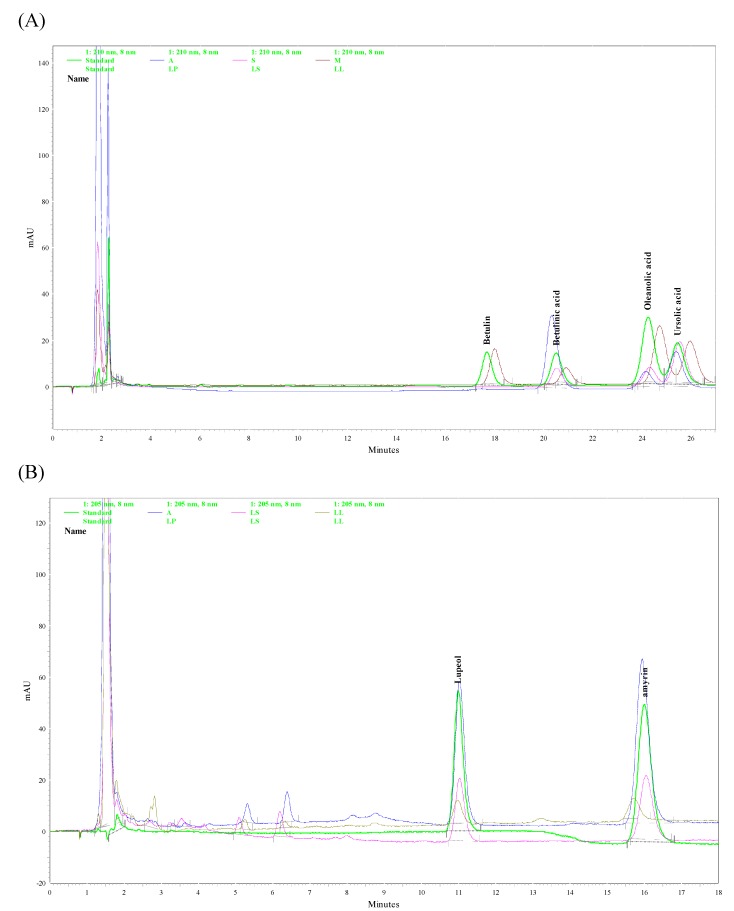
HPLC chromatograms of *Ligustrum* plants. (A) Detector responses at 210 nm. (B) Detector responses at 205 nm. Green trace: Standard, Blue trace: *L. pricei* (LP), Pink trace: *L. sinens* (LS), Brown trace: *L. lucidum* (LL).

According to phytochemical reports, LL contained oleanolic acid and ursolic acid [9] which possess anti-inflammatory activity and are suggested as the major active components of LL for its hepatoprotective and antidiabetic effects [1,3,4]. However, some triterpenoid compounds, such as amyrin, betulin, betulinic acid and lupeol, also possess anti-inflammatory activity [18,19,20,21]. Finally, we assayed of the methanol extracts from *Ligustrum* plants leaves for the six above-mentioned triterpenoid by HPLC-PAD. Their HPLC chromatographs were shown in Figure 6. Table 2 shows the triterpenoid contents in the methanol extract from the *Ligustrum* plants leaves measured with HPLC-PDA. We confirmed that the plant leaves of the three *Ligustrum* spp. also contained oleanolic acid and ursolic acid, which are two common triterpenoids in *Ligustrum* plants [9]. The highest contents of oleanolic acid and ursolic acid were observed in LL followed by LP and LS. In addition to the above-mentioned triterpenoid compounds, amyrin, betulinic acid and lupeol were first found and quantified in these three *Ligustrum* plants leaves. The highest contents of amyrin and lupeol were observed in LP followed by LS and LL. LP also had the highest content of betulinic acid compared to LL and LS. 

Betulin was only detected in LL. Therefore, there are the differential amounts of triterpenoids in the methanol extracts from these three *Ligustrum* plants leaves. The difference in the amounts of triterpenoids may be related to the analgesic/anti-inflammatory properties of the methanol extracts from these three *Ligustrum* plants leaves. We further suggested that amyrin, betulinic acid and lupeol are the major active components of LP for anti-inflammatory effects. The major active components of LL for anti-inflammatory effects are oleanolic acid and ursolic acid, in consistence with other reports on the hepatoprotective and antidiabetic effects [1,3,4]. Besides, there are some different peak groups at 2-10 min in 205 nm HPLC chromatograph between LL and other two *Ligustrum* plants (LP and LS) (Figure 6B). This result showed that some different nonpolar compounds existed in three collected *Ligustrum* plants, and these unidentified peaks and our identified triterpenoids may use to distinguish three collected *Ligustrum* plants. However, these unidentified peaks must be identified and their pharmacological activities also must be clarified in the future.

**Table 2:** The yield and triterpenoid contents of *Ligustrum* plants leaves extracted with methanol.

Plants	Yield (%)	Amyrin (μg/g)	Betulin (μg/g)	Betulinic acid (μg/g)	Lupeol (μg/g)	Oleanolic acid (μg/g)	Ursolic acid (μg/g)
*L. pricei*	16.34	3782.81 ± 38.83*	-	1877.89 ± 5.82***	3765.78 ± 61.69*	380.21 ± 15.63**	1070.76 ± 5.90**
*L. sinense*	28.74	2031.93 ± 24.31	-	313.43 ± 7.53	1983.26 ± 36.71	205.12 ± 0.30	680.25 ± 14.21
*L. lucidum*	41.77	603.86 ± 5.99***	623.63 ± 3.32	472.26 ± 1.72**	629.68 ± 11.06*	957.69 ± 4.81***	3412.53 ± 6.84***

Data were expressed as mean ± SEM for three repeats. * *p* < 0.05, ** *p* < 0.01, *** *p* < 0.001 compared with *L. sinense*.

## 3. Experimental

### 3.1. Preparation of plant extracts and drugs

The aerial parts of LP (no. ICPS-L20050131001) were sampled from the Chi-Tou Forest Recreational Area in Nantou County (Taiwan). The aerial parts of LS (no. ICPS-L20050221001) were sampled from the Botanical Garden of the National Museum of Natural Science, Taichung City. The aerial parts of LL (no. ICPS-L20050123001) were sampled from the Herbal Garden of China Medical University at Taichung City. They were identified by Professor Dr. Chung-Chuan Chen of the Department of Chinese Medicinal Resources, College of Pharmacy, China Medical University and deposited in the herbarium of the Graduate Institute of Chinese Pharmaceutical Sciences, China Medical University. The dried leaves (100 g) obtained from the *Ligustrum* plants were extracted five times with methanol. The resultant extract was combined and concentrated under reduced pressure to obtain the methanol extract. The yield of *Ligustrum* plants leaves is shown in Table 2. The methanol extract from the *Ligustrum* plants leaves (0.1, 0.25, 1 g/kg) was dissolved in 0.5% carboxymethylcellulose and administered orally 60 min prior to the injection of the inducer. Acetylsalicylic acid (ASA, 300 mg/kg) and indomethacin (Indo, 10 mg/kg) were also prepared as suspension with 0.5% carboxymethylcellulose and administered orally 60 min prior to the injection of the inducer. For the *in vitro* cyclooxygenase inhibition activity assay, the methanol extract from the *Ligustrum* plants leaves was dissolved in 50 mM phosphate buffer (pH 7.4).

### 3.2. Animals

Male Sprague /Dawley rats, weighing 200-250 g, were used for the study of anti-inflammatory activities and in the microvascular permeability test. Male ICR mice, weighing 20-25 g, were used for the testing of analgesic effects. All animals were used in accordance to the Guiding Principles of the Care and Use of Laboratory Animals of the China Medical University. They were housed for at least one week before starting the experiment with free access to standard food pellets (supplied and designed by Fwusow Industry Co. LTD., Taiwan) and tap water and housed in a regulated environment (23 ± 1 °C temperature and 60% humidity), wherein a 12-12 h light/dark cycle (light phase: 08:00-20:00 h) was maintained. Drugs were administered and the analgesic, anti-inflammatory and microvascular permeability assays were performed using the double-blind method. After behavioral measurement, all animals were euthanized with carbon dioxide.

### 3.3. Acetic acid-induced abdominal writhing response in mice

This method is described in our previous report [12]. Briefly, the writhing response was induced by intraperitoneal injection of 1% acetic acid (v/v, 10 ml/kg body weight). Three different doses of the methanol extract from *Ligustrum* plant leaves (0.1, 0.25, 1 g/kg) were orally administered to mice 60 min before acetic acid injection. Five minutes after the injection of acetic acid, the writhing number per mouse was counted for 10 min during acetic acid-induced abdominal writhing responses [5]. Control animals received a vehicle solution in the same experiments. The writhing number permitted us to express the percentage of protection using the following ratio: (control mean-treated mean) / control mean × 100.

### 3.4. Formalin-induced licking response in mice

This method is described in our previous report [12] with modification from Shibata *et al.* [6]. Briefly, pain was induced by injecting 25 μL of 1% formalin (v/v) into the right subplantar hind paw. The methanol extracts from *Ligustrum* plant leaves (0.1, 0.25, 1 g/kg) were orally administered to mice 60 min before formalin injection. The two distinct periods of the licking and biting the injected paw after the injection of formalin was observed. The first period (early phase) was recorded at 0-5 min and the second period (late phase) was recorded at 10-35 min [12]. The time(s) spent licking the injected paw was measured as an indicator of pain response.

### 3.5. Carrageenan-induced paw edema in rats

The carrageenan-induced paw edema model was described in our previous report [12] with modification from Winter [7]. Briefly, rats were injected 0.1 mL of 1% carrageenan into the right hind foot under the plantar aponeurosis. The paw volume of each animal was determined (Vt) 30, 60, 90, 120, 150, 180, 210 and 240 min after carrageenan injection. Paw volume was averaged with three measurements in each period using a plethysmometer (7150 Ugo Basile) that did not differ by more than 4%. The edema percentage at each record was calculated by comparing the average volume of the hind paws of each animal (Vt) after the injection of carrageenan with the average volume of the hind paws of each animal (Vo) before any treatment [12]. Inhibition percentages were obtained for each group by using the following ratio: [(Vt/Vo)control−(Vt/Vo)treated] / (Vt/Vo)control × 100.

### 3.6. Microvascular permeability test in rats

The microvascular permeability test was described in our previous report [12]. Briefly, rats were anesthetized and their abdominal skin was marked with eight 2-cm diameter circles 30 min after treatment of the methanol extract of LP leaves. The bacterial toxins, LPS (500 μg/site) and LTA (250 μg/site), or inflammatory mediators, such as serotonin (1 nM), histamine (10 μM), bradykinin (10 nM) and SNP (200 nM) were injected into the central area of the eight circle on the abdominal skin after intravenous injection of 20 mg/kg Evan’s blue dye. After 1 hour, all rats were sacrificed and the stained skin of the injected site was excised. These stained skins were infiltrated with 300 μL sodium sulfate and 700 μL acetone overnight to extract the abdominal Evan’s blue extravasations. The infiltrated solutions were centrifuged at 2,000 ×g for 20 min, and the supernatants were collected and transferred into a 96-well plate to measure the absorbance at 620 nm [12]. The alternation of vascular permeability was measured for each group using the following ratio: (A_induced_ - A_saline_) / A_saline_ × 100, where A_induced_ is the absorbance of Evan’s blue extravasation in the circle treated with bacterial toxins or inflammatory mediators and A_saline_ is the absorbance of Evan’s blue extravasation in the circle treated with saline.

### 3.7. Cyclooxygenase-2 inhibiting activities assay in vitro

The cyclooxygenase inhibiting activities of the methanol extract from *Ligustrum* plants leaves were assayed using a cyclooxygenase inhibitor screening kit (Cayman No. 760111). One-hundred fifty microliters of assay buffer and 10 μL of heme were loaded to each well followed by the addition of 10 μL of cyclooxygenase-2 solution or assay buffer and 10 μL of 50 mM phosphate buffer or the methanol extract solution from *Ligustrum* plants leaves. After a 5-min incubation at room temperature, 20 μL of TEMP and arachidoic acid were added to each well. The reactive mixture was incubated for 5 min at room temperature and put into a Bio-Teck PowerWave 340X microplate reader to record the absorbance at 590 nm [22].

### 3.8. Determination of triterpenoids by HPLC-PDA

The methanol extract from the *Ligustrum* plants leaves was dissolved in methanol and filtered with a 0.22-μm filter. A Shimadzu HPLC VP series system and Shimadzu Class-VP^TM^ chromatography data system were used for this measurement. The analytical condition for betulin, betulinic acid, ursolic acid and oleanolic acid was consistent with our previous report [12]. A Supelco Discovery® C18 (150 × 4.6 mm, 5 μm) column (Sigma-Aldrich Co., St. Louis, MO, USA) was also used for separating amyrin and lupeol. The mobile phase for amyrin and lupeol was a mixture of methanol and water (97:3, v/v) at a flow rate of 1 mL/min. The chromatographic peaks of the six common triterpenoids were confirmed by comparing their retention times and UV spectra.

### 3.9. Statistical analysis

All data obtained during the analgesic, anti-inflammatory and microvascular permeability assays are expressed as the mean ± standard errors (SE), and were analyzed using a one-way analysis of variance (ANOVA) followed by Scheff’s test. When the probability (*p*) was less than 0.05, the difference was considered significant.

## 4. Conclusions

In conclusion, LP is a potential analgesic and anti-inflammatory plant among the three *Ligustrum* medicinal plants used in traditional Chinese medicine. Its analgesic and anti-inflammatory dose is from 0.25 g/kg and lower than other report of *L. robustum* (0.5~1.7 g/kg) and LL [2,10,11]. 

**Figure 7:**
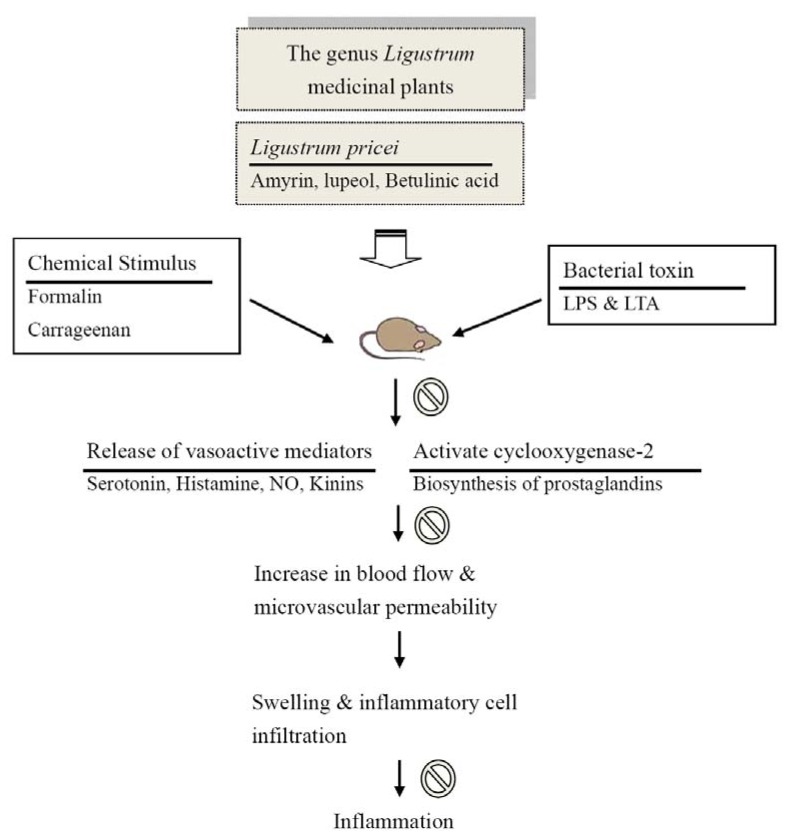
The proposed biological action of *Ligustrum* plants, especial *Ligustrum pricei,* as a potential anti-inflammatory plant. Prohibition sign indicates that the inhibitory effect of *Ligustrum pricei*.

Among the six triterpenoids, the highest contents of amyrin, betulinic acid and lupeol were found in LP*.* Several researchers have pointed out that amyrin (5-10 mg/kg) possesses anti-inflammatory effects via the inhibition of prostaglandins and TNFα using the NF-kappaB and CREB signalling pathways [20,23]. Lupeol (10-50 mg/kg) possesses anti-inflammatory activity via a reduction of cell infiltration and the prevention of the production of some pro-inflammatory mediators, such as prostaglandins and cytokines [21,24]. Betulinic acid (5-20 mg/kg) has anti-inflammatory actions and potential as inhibitor of phospholipase A_2_ [19]. From our present results and the pharmacological reports of triterpenoids and other *Ligustrum* plants, we suggested that amyrin, betulinic acid and lupeol are three of the active components of LP because the anti-inflammatory potency of LP is equivalent with its triterpenoid contents and the anti-inflammatory potency of these triterpenoids. The analgesic and anti-inflammatory mechanism of LP might be partially related to the modulation of microvascular permeability via the inhibition of inflammatory mediators, including autocrines, kinins, nitric oxide and prostaglandins, and its inhibitory activity against cyclooxygenase-2 (Figure 7). The role of pro-inflammatory cytokines, NF-kappaB and CREB signalling pathways on the anti-inflammatory activity of LP requires further investigation.
